# Gender-related differences in career development among gynecologic oncology surgeons in Europe. European Network of Young Gynecologic Oncologists’ Survey based data

**DOI:** 10.3389/fonc.2022.1005130

**Published:** 2022-12-07

**Authors:** Tanja Nikolova, Michaela Bossart, Joanna Kacperczyk-Bartnik, Zoia Razumova, Alexandra Strojna, Nicolò Bizzarri, Andrei Pletnev, Natalia R. Gómez-Hidalgo, Charalampos Theofanakis, Maximilian Lanner, Ilker Selcuk, Alexander Shushkevich, Chelariu-Raicu Anca, Natasha Nikolova, Nicole Concin, Kamil Zalewski

**Affiliations:** ^1^ Klinikum Mittelbaden, Academic Teaching Hospital of Heidelberg University, Baden-Baden, Germany; ^2^ Department of Obstetrics and Gynecology, St. Josefskrankenhaus, Freiburg, Germany; ^3^ 2nd Department of Obstetrics and Gynecology, Medical University of Warsaw, Warsaw, Poland; ^4^ Department of Women’s and Children’s Health, Karolinska Institute, Stockholm, Sweden; ^5^ Department of Gynecology and Gynecologic Oncology, Kliniken Essen-Mitte, Essen, Germany; ^6^ Unità Ginecologia Oncologica, Dipartimento per la salute della Donna e del Bambino e della Salute Pubblica, Fondazione Policlinico Universitario a Gemelli, Rome, Italy; ^7^ Department of Obstetrics and Gynecology, University of Zielona Gora, Zielona Gora, Poland; ^8^ Gynecological Oncology Department, Vall d’Hebron Hospital, Autonoma University of Barcelona, Barcelona, Spain; ^9^ Department of Gynecological Oncology, General Hospital of Athens Alexandra, Athens, Greece; ^10^ Department of Gynecology, Private Hospital Villach, Villach, Austria; ^11^ Department of Gynecologic Oncology, Ankara City Hospital, Ankara, Turkey; ^12^ Department of Surgery, Institute of Biology and Medicine, Taras Shevchenko National University of Kyiv, Kyiv, Ukraine; ^13^ Department of Obstetrics and Gynecology, University Hospital, Ludwig-Maximillians-University, Munich, Germany & German Cancer Consortium and German Cancer Research Center, Heidelberg, Germany; ^14^ Center of Perinatal and Reproductive Medicine, University of Perugia, Perugia, Italy; ^15^ Department of Gynecology and Obstetrics, Innsbruck Medical University, Innsbruck, Austria & Department of Gynecology and Gynecologic Oncology, Kliniken Essen-Mitte, Essen, Germany; ^16^ Department Gynecologic Oncology, Holycross Cancer Center, Kielce, Poland

**Keywords:** gender-related differences, gender inequalities, gynecologic oncology surgeons, career development, leadership, salary

## Abstract

**Introduction:**

Gender-related differences in career development are well known issues in various professions. An international survey on gender-related differences was performed among young gynecologic oncology surgeons in Europe to identify potential gender inequalities in career development.

**Material and methods:**

A survey on demographics, clinical and academic working environment, family/parenting, career development, salary and leadership was sent to all members of the European Network of Young Gynecologic Oncologists (ENYGO), which is a network within the European Society of Gynecologic Oncology (ESGO). Gynecologic oncology surgeons and obstetricians/gynecologists who actively work in this field in Europe were included in the study.

**Results:**

Responses were analyzed from 192 gynecologic oncology surgeons of whom 65.1% (125/192) were female (median age 37, IQR: 34 - 42) and 34.9% (67/192) were male (median age 38, IQR: 36 - 41). Male reported to perform a median of 15 and female a median of 10 operations per month *(p = .*007). Among female, 24.8% had a leadership position vs. 44.8% among male, crude *OR* = 2.46, 95% *CI* 1.31-4.62, *p<*.01. When stratifying for age under 41 and having children, 36.7% of male and 5.6% of female had a leadership position, adjusted *OR* 10.8, 95% *CI* 3.28-35.64, *p*<.001. A significantly higher proportion of female compared to male believed they earned less than their gender counterparts at the same clinical position and with same qualifications (30.4% vs. 2.5%, *p<*.001). There was not a statistically significant gender difference in the academic qualification PhD degree or professorship (*p* = .92 and *p* = .64, respectively). In the previous year, male published more peer-reviewed articles than female (median 3 vs. median 2; *p* = .017).

**Conclusion:**

This first comprehensive survey on gender-differences in gynecologic oncology in Europe revealed that there are gender gaps concerning several aspects during the critical time of career development in the young generation of gynecologic oncology surgeons. These gender gaps are particularly reflected by a lower rate of female leadership positions. ENYGO and ESGO are dedicated to work on solution to overcome the identified obstacles and to support closing gender gaps.

## Introduction

According to the report of the Organization for Economic Cooperation and Development countries (OECD), almost half of all medical doctors in 2019 were female (1). Female accounted for more than half of students entering PhD programs in the United States (2). Based on membership data of the European Society of Gynecological Oncology (ESGO), the proportion of female gynecologic oncologists has been constantly increasing in the last decade. While in 2009 more than two thirds of members were male, the gender distribution reached parity in 2019. An even faster growing trend is evident among the younger generation of gynecologic oncologists in Europe. According to the membership directory of the European Network of Young Gynecologic Oncologists (ENYGO), which is a network of ESGO comprising the young generation of the gynecologic oncology surgeons, 2/3 of members were female in 2019.

On the other side the published literature indicates that in the field of medicine female still face manyfold career barriers in comparison to their male colleagues. Female are reported to have less opportunities to research and to publish, are promoted more slowly and at a lower rate e.g. for professorship positions and are less paid than their male counterparts (3–9). Studies performed among medical professionals in different fields show that although female participation in medical schools and hospitals is increasing, male professionals still dominate at senior leading positions (10, 11).

To our knowledge there is not any published data on gender-related differences in career development among gynecologic oncology surgeons in Europe.

We performed a comprehensive survey consulting the young generation of gynecologic oncology surgeons and specialists in gynecology and obstetrics with a major focus on gynecologic oncology surgery, who are working in academic and nonacademic surgical centers. The aim of this survey was to investigate potential gender differences and potential obstacles during the time of career development.

## Methods

A survey consisting of 85 questions was designed and approved by the Institutional Review Board at the Medical School, University in Kielce, Poland (Number: 65/2021). The questionnaire consisted of questions related to demographics; clinical and academic working environment; family and parenting; career development; leadership including clinical director, head of department and head of division, as well as salary. The questions validity was thereafter assessed through a review of 12 ENYGO members who were selected to represent the different sociodemographic characteristics (gender, age, nationality, country of practicing, partner status, children, and sexual orientation). Each reviewer assessed the questions and response options to ensure their clearness and inclusivity with regards to diverse life and professional experiences.

ENYGO is a network within ESGO, which comprises of subspecialists in gynecologic oncology surgery who are ≤40 years of age, and of fellows in subspeciality training for gynecologic oncology surgery (all fellows in training independent of age), as well as of specialists in gynecology and obstetrics ≤40 who are actively working in the field of gynecologic oncology surgery. The survey was administrated anonymously to all ENYGO members in electronic format *via* the survey tool “SurveyMonkey” (SurveyMonkey Inc. Palo alto, CA, USA).

The first participating request was sent in October 2019. It was promoted during the ESGO conference in Athens in November of 2019 and after that, launch was repeated in January and February of 2020. The online software allowed respondents to complete the survey without answering all the questions, hence each question was not necessarily answered by all the respondents.

Inclusion criteria for distribution of this survey were ENYGO members who were practicing in Europe at the time of survey. Respondents who were not actively working in the field of gynecologic oncology surgery and were not practicing in Europe were excluded from the study.

### Statistical analysis

Statistical data analysis was performed using statistical software SPSS version 26. Descriptive statistics was used to summarize the results of the questionnaire. As not all the questions were answered by all the respondents, the percentage was calculated using a number of respondents to the specific question as a denominator. Categorical and ordinal data associations were tested using Chi-squared or Fisher’s exact test and Mann-Whitney U test. The Cochran-Mantel-Haenszel test of homogeneity was used in cases where controlling for gender or age with other significant factors had to be explored. All tests were two-tailed at the level of statistical significance α = 5%.

## Results

### Description of the study population

The survey link was sent to 745 ENYGO members. A total of 230 recipients replied after the survey was launched by the third time (response rate of 30.9%). Of them, 38 (16.5%) respondents were excluded, as they did not meet the inclusion criteria (31 were not working in Europe, 7 were not actively working in the field of gynecologic oncology surgery). Thus, answers from a total of 192 respondents were included in the final analysis with a median age 37 years (IQR: 35 - 42). Of them, 132 (68.6%) were gynecologic oncology surgeons, either subspecialists who have completed their training (*n*=84, median age 40 years, IQR: 36 - 44) or fellows in training (*n*=48, median age 36 years, IQR: 33 - 38), and 60 (31.4%) were specialists in gynecology and obstetrics who were actively working as surgeons in gynecologic oncology (median age 37 years, IQR: 34 - 42). **Table 1** shows the general demographic characteristics of the respondents.

**Table 1:** Demographic characteristics.

	Male		Female		Total	
	*n*	%	*n*	%	*n*	%
**Gender**	67	34.9%	125	65.1%	192	
**Age**						
≤ 30	0	0.0%	17	13.6%	17	8.9%
31-40	49	73.1%	72	57.6%	121	63.0%
41-50	11	16.4%	30	24.0%	41	21.4%
51-60	5	7.5%	6	4.8%	11	5.7%
> 61	2	3.0%	0	0.0%	2	1.0%
**Marital Status**						
Single	4	8.3%	20	23.0%	24	17.8%
Married/partner	43	89.6%	64	73.6%	107	79.3%
Divorced	1	2.1%	3	3.4%	4	3.0%
**Children**						
Yes	38	79.2%	57	64.0%	95	69.3%
No	10	20.8%	32	36.0%	42	30.7%
**Number of Children**						
0	29	43.3%	68	54.4%	97	50.5%
1	13	19.4%	29	23.2%	42	21.9%
2	17	25.4%	22	17.6%	39	20.3%
≥ 3	8	11.9%	6	4.8%	14	7.3%
**Parental leave**						
Yes	25	75.8%	53	96.4%	78	88.6%
No	8	24.2%	2	3.6%	10	11.4%

### Clinical working environment and leadership

The obtained data about the clinical working environment are presented in **Table 2**.

**Table 2:** Clinical characteristics.

	Male			Female			Total		p - value
	*n*	%		*n*	%		*n*	%	
**Working in academic oncology center**									.76
Yes	42	85.7%		72	83.7%		114	84.4%	
No	7	14.3%		14	16.3%		21	15.6%	
**Level of training**									.59
Fellow in Gyn. Onc.	14	20.9%		34	27.2%		48	25.0%	
Gyn. Onc. Surgeon	32	47.8%		52	41.6%		84	43.8%	
Specialist in Ob/Gyn.	21	31.3%		39	31.2%		60	31.3%	
**Number of operative cases per month**									.006
< 5	1	2.2%		9	10.5%		10	7.5%	
5-10	9	19.1%		33	38.4%		42	31.6%	
> 10	37	78.7%		44	51.1%		81	60.9%	
**Number of night shifts per month**									.64
0	4	8.5%		9	10.7%		13	9.9%	
1-4	19	40.4%		42	50.0%		61	46.6%	
5-9	18	38.3%		25	29.8%		43	32.8%	
≥ 10	6	12.8%		8	9.5%		14	10.7%	

There was a significant difference in the number of reported operative cases per month between male and female. Male reported a median of 15 and female a median of 10 cases per month (*U (N_male_* = 29, *N_female_* = 71) = 677*, z* = -2.71*, p = .*007, (η*^2^ = .*072). The number of night shifts per month did not differ between male and female (median of 4 for both genders).

The data about the reported leadership positions in male versus female, as well as the subsequent stratifications are presented in **Table 3**.

**Table 3:** Leadership position analysis.

Male	Female	Crude OR (95% *CI*)	p - value	χ^2^	Effect size (ϕ)		
44.8% (30/67)	24.8% (31/125)	2.46 (1.31-4.62)	.005	(1, N=192) = 8.03	.205
**Stratified**							
Male	Female	Adjusted OR (95% *CI*)	p - value	$\chi_{\text{CMHtest}}^{2}$	p - value	$\chi_{\text{BDtest}}^{2}$	p - value
“Being married/living with a partner”							
48.8% (20/41)	17.5% (11/63)	3.89 (1.75-8.65)	.001	(1, *N* = 139) = 11.6	.001	(1, *N* = 139) = 0.64	.425
“Being married to a medical doctor/living with a partner who is a medical doctor”							
45.8% (11/24)	24.3% (9/37)	3.37 (1.46-7.75)	.004	(1, *N* = 115) = 8.58	.003	(1, *N* = 115) = 0.46	.496
Age under 41 years							
32.7% (16/49)	13.5% (12/89)	3.11 (1.53-6.36)	.002	(1, *N* = 192) = 10.3	.001	(1, *N* = 192) = 0.001	.993
Parental status (having children vs. not having)							
50% (19/38)	19.3% (11/57)	3.91 (1.75-8.73)	.001	(1, *N* = 137) = 11.6	.001	(1, *N* = 137) = 0.11	.74
Age under 41 and having children							
36.7% (11/30)	5.6% (2/36)	10.8 (3.28-35.64)	<.001	(1, *N* = 137) = 19.3	<.001	(3, *N* = 137) = 3.77	.29

There was a significant difference in reported leadership positions between male and female. 44.8% (30/67) of male compared to 24.8% (31/125) of female reported to have a leadership position, (crude *OR* 2.46, 95% *CI* 1.31-4.62, *p* = .005, χ^2^ (1, *N* = 192) = 8.03, small effect size ϕ = .205).

When stratified for age under 41 and having children, the gender gap was the most pronounced with 36.7% (11/30) of male compared to 5.6% (2/36) of female having a leadership position (adjusted *OR* 10.8, 95% *CI* 3.28-35.64, *p*<.001, $\chi_{\text{CMHtest}}^{2}$ (1, *N* = 137) = 19.3, *p*<.001, $\chi_{\text{BDtest}}^{2}$ (3, *N* = 137) = 3.77, *p* = .29).

Among respondents with children, 96.4% (53/55) of female and 75.8% (25/33) of male used parental leave. 58.5% (31/53) of female used parental leave for a year or less (6 weeks to 12 months) and 41.5% (22/53) for more than a year (up to a max of 3 years). 66.7% (16/24) of male used parental leave for a maximum of six weeks (1 to 6 weeks) and 33.3% (8/24) for more than six weeks (up to a maximum of one year). Among both genders, there was no association between the leadership position and the length of parental leave (for female: χ^2^ (1, *N* = 53) = 0.298, *p* = .56; for male: χ^2^ (1, *N* = 24) = 2.1, *p* = .15).

**Figure 1:**
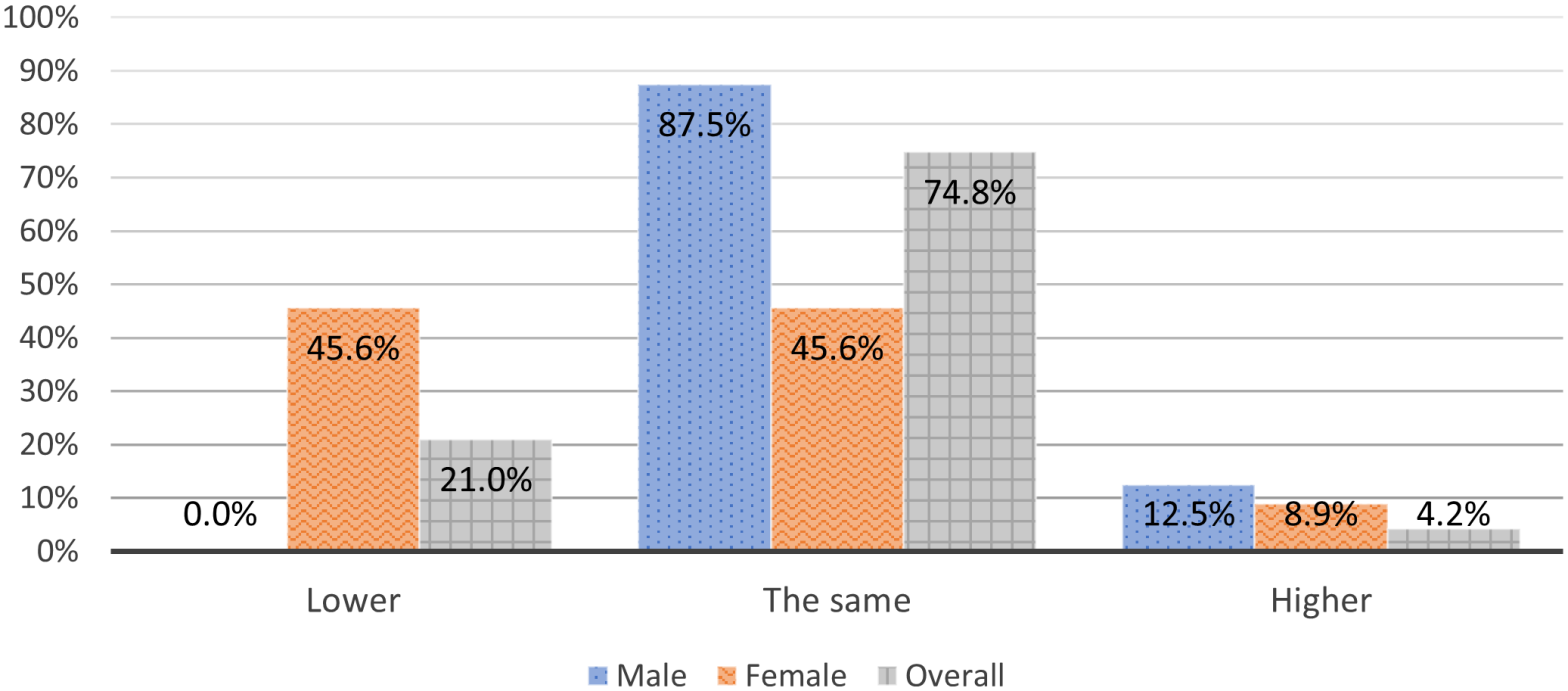
Is your achieved clinical position same, higher or lower in comparison to your opposite gender colleagues with the same experience and at approximately same age?

The data on the question: “Is your achieved clinical position same, higher or lower in comparison to your opposite gender colleagues with the same experience and at approximately same age?” is presented in **Figure 1**. There was a significant medium to strong association between the perception of having achieved the “adequate” clinical position and gender. 45.6% (36/79) of female believed that their achieved clinical position was lower than the one of their male counterparts with approximately the same experience and age, 45.6% (36/79) believed it was the same, and 8.9% (7/79) of female believed that their position was higher. On the other side, 87.5% (35/40) of male believed that their achieved clinical position was the same as their female counterparts with approximately same experience and age, 12.5% (5/40) believe that it was higher, and none believed that their position was lower (χ^2^ (2, *N* = 119) = 26.4, *p*<.001, medium to strong effect size *V* = .471).

On the question: “Are you happy with your current clinical achievements?”, more than half of male respondents, i.e. 53.8% (21/39), declared that they were happy, and 46.2% (18/39) declared that they were not happy with their current clinical achievements, while 32.5% (26/80) of female respondents declared their happiness and more than half, i.e. 67.5% (54/80), their unhappiness with the current clinical achievements (*OR* 2.42, 95% *CI* 1.11-5.31, *p* = .025, χ^2^ (1, *N* = 119) = 4.99, small effect size ϕ = .205).

30.4% (24/79) of female and 2.5% (1/40) of male believed that their salary was lower in comparison with their gender counterparts at the same position and clinical/academic qualifications, while 97.5% (39/40) of male and 69.6% (55/79) of female believed that their salary was the same or higher (*OR* 17.01, 95% *CI* 2.21-131.14, *p*<.001, χ^2^ (1, *N* = 119) = 12.44, medium effect size ϕ = .323).

### Academic working environment

Data about the academic working environment are presented in **Table 4**.

**Table 4:** Academic characteristics.

	Male		Female		Total		p - value
	*n*	%	*n*	%	*n*	%	
**Academic qualifications:**							.59
None	13	26.5%	30	34.5%	43	31.6%	
PhD	22	44.9%	37	42.5%	59	43.4%	
Professor	14	28.6%	20	23.0%	34	25.0%	
**Professorship:**							.11
Assistant Professor	3	21.4%	8	40.0%	11	32.4%	
Associate Professor	10	71.4%	7	35.0%	17	50.0%	
Full Professor	1	7.2%	5	25.0%	6	17.6%	
**Did you have publications in the previous year?**							.30
Yes	36	78.3%	60	69.8%	96	72.7%	
No	10	21.7%	26	30.2%	36	27.3%	
**Number of full-text publications as first and last author in your medical career:**							.024
0	17	37.0%	35	40.7%	52	39.4%	
1-3	18	39.1%	45	52.3%	63	47.7%	
4-6	10	21.7%	4	4.7%	14	10.6%	
> 6	1	2.2%	2	2.3%	3	2.3%	
**Number of medical conferences (national and international) attended in the previous year:**							.13
≤ 1	5	10.2%	21	24.1%	26	19.1%	
2-3	30	61.2%	47	54.0%	77	56.6%	
≥ 4	14	28.6%	19	21.8%	33	24.3%	
**Number of conference presentations (oral and poster) in the previous year:**							.012
0	6	13.0%	27	31.0%	33	24.8%	
1-4	29	63.0%	54	62.1%	83	62.4%	
5-9	8	17.4%	5	5.7%	13	9.8%	
≥ 10	3	6.5%	1	1.1%	4	3.0%	
**Medical grants/funding in the previous 5 years:**							.60
Yes	22	55.0%	48	60.0%	70	38.3%	
No	18	45.0%	32	40.0%	50	41.7%	

55.2% (37/67) of female respondents and 62.9% (22/35) of male respondents hold a PhD title (χ^2^ (1, *N* = 102) = 0.55, *p* = .92, adjusted for Bonferroni correction). More male, 51.9% (14/27), than female, 40% (20/50), reported to have a professor position. However, this difference was not statistically significant (χ^2^ (1, *N* = 77) = 0.99, *p* = .64, adjusted for Bonferroni correction).

Parental leave duration was no adverted factor for female to hold an academic qualification (PhD degree and/or professorship; χ^2^ (2, *N* = 52) = 2.71, *p = .*258).

There was a significant small to intermediate effect between number of peer reviewed publications in the previous year and the gender. On average, male had more publications (median = 3) in the previous year than female (median = 1). The difference was significant *U (N_male_* = 46, *N_female_* = 86) = 1,488*, z* = -2.38*, p = .*017, η*^2^ = .*042. When stratified for having children, the small to intermediate effect between number of publications in the previous year and gender remained. Male with children published more (median = 2) than female with children (median = 1) in the previous year. The difference was significant *U (N_male_* = 36, *N_female_* = 55) = 741.5*, z* = -2.06*, p = .*04, η*^2^ = .*043.

A comparable number of males and females have received at least one medical grant/funding in the previous five years 45% (18/40) of male vs. 40% (32/80) of female (χ^2^ (1, *N* = 120) = 0.247, *p* = .60).

There was a significant intermediate effect between number of congress presentations in the previous year and gender. On average, male had more congress presentations (median = 3) than female (median = 2). The difference was significant *U* (*N_male_* = 46, *N_female_* = 87) = 2,651.5, *z* = 3.13, *p* = .002, η*^2^* = .071.

On the question: “Are you happy with your current academic achievements?”, 27.3% (15/55) of female and 41.9% (13/31) of male confirmed their happiness with their current academic achievements, this association was not statistically significant (χ^2^ (1, *N* = 86) = 1.94, *p* = .164).

### Challenges for career development and barriers for gender parity

Among female, child planning was extremely important in 31.5% (17/54) of the respondents, considerably important in 37% (20/54) and not important at all in 13% (7/54), while among male it was extremely important in 7.5% (3/40) of the respondents, considerably important in 32.5% (13/40) and not important at all in 20% (8/40). Planning parenting was playing a major role in carrier development and there was a moderate positive correlation with gender (*d* = .35, *p* = .001).

On the question: “Do you think that the parental leave has affected your clinical career?”, 48.1% (26/54) of female deemed that parental leave has affected their clinical carrier vs. 20% (4/20) of male (*OR* 3.71, 95% *CI* 1.11-12.57, *p* = .029, χ^2^ (1, *N* = 74) = 4.79, small to moderate effect size ϕ = .26). When asked in which way has the parental leave affected their clinical career, 45.7% (21/46) of female stated that it was the lack of surgical activities while on leave, and 39.1% (18/46) assessed that due to the parental leave they have missed their career advancements.

On the question: “If the parental leave affected your academic career?”, there was again a significant difference between male and female. 44.4% (24/54) female and 5.9% (1/17) man perceived that parental leave had adversely affected their academic career (*OR* 12.8, 95% *CI* 1.58-103.53, *p *= .004, χ^2^ (1, *N* = 71) = 8.43, moderate effect size ϕ = .35). 42.5% (17/40) female stated that because of parental leave, they did not manage to actively participate in research projects and could not publish, 27.5% (11/40) stated that they did not get the desired academic position, 12.5% (5/40) stated that they did not have time to enroll in PhD studies and did not have time to work with students/fellows/residents.

A significant higher number of female 41.8% (23/55) than of male 14.3% (3/21) were feeling underestimated by their manager, because of using parental leave (*OR* 4.31, 95% *CI* 1.14-16.4, *p* = .024, χ^2^ (1, *N* = 76) = 5.12, small to moderate effect size ϕ = .26).

**Figure 2:**
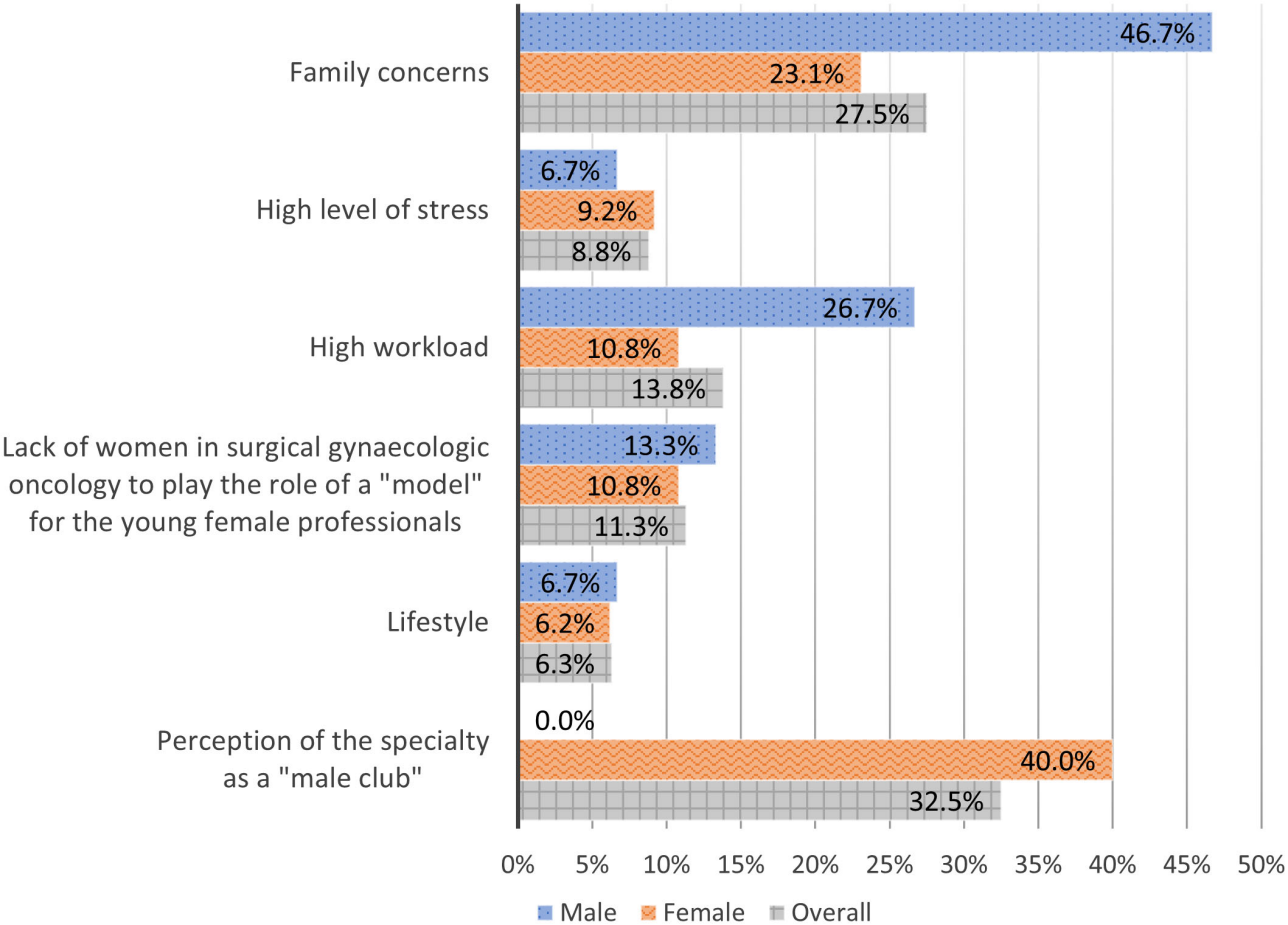
Perceived obstacles for career success among female in surgical gynecologic oncology.

The significant majority of female, 79.3% (65/82), see obstacles for career success for females in surgical gynecologic oncology compared to less than half of male, 46.2% (18/39), (*OR* 4.46, 95% *CI* 1.95-10.2, *p*<.001, χ^2^ (1, *N* = 121) = 5.12, moderate effect size ϕ = .33). **Figure 2** presents the perceived obstacles for career success among female in surgical gynecologic oncology.

### Suggested ways for gender parity achievement

**Figure 3:**
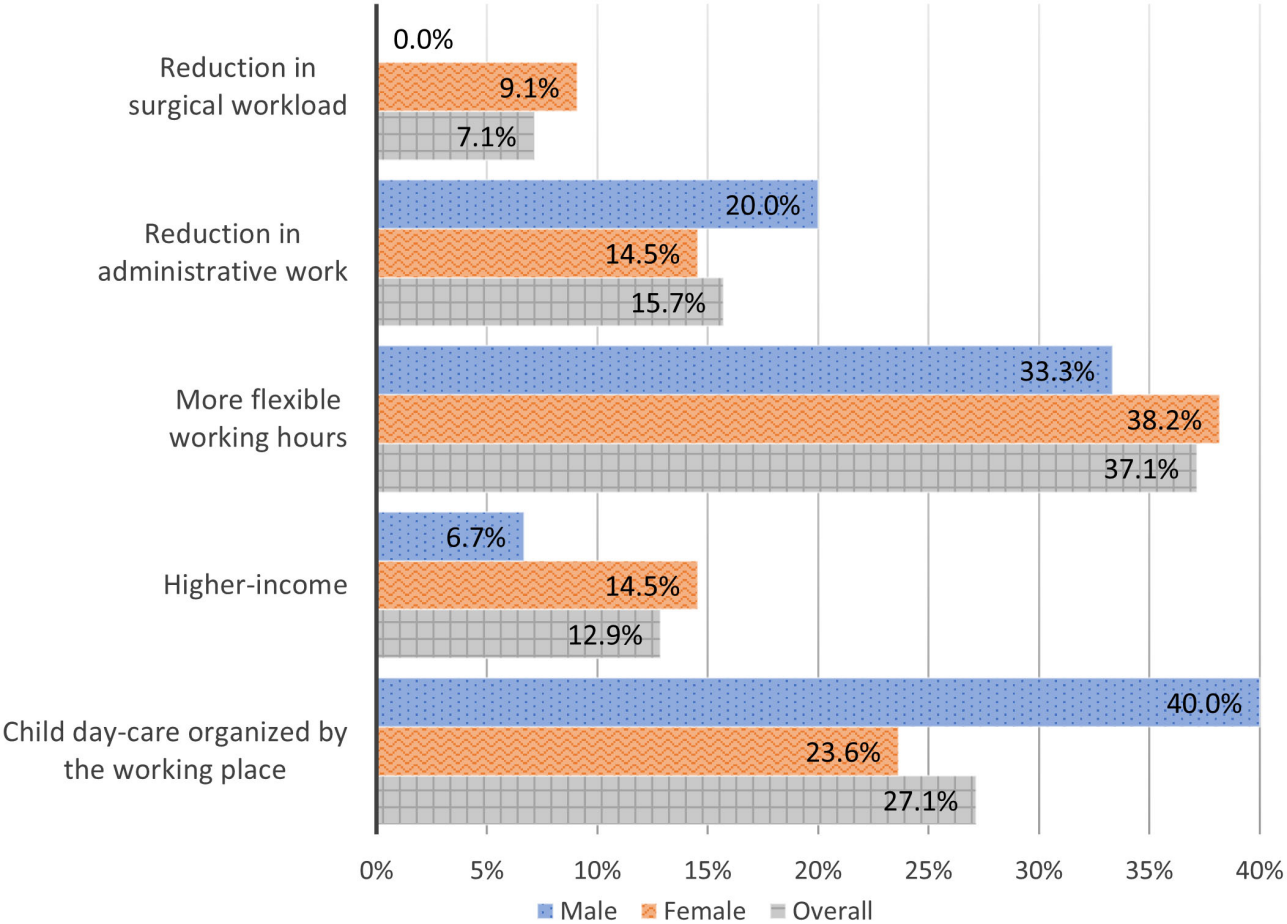
Suggested ways to increase the feasibility for a woman to be both a successful gynecologic oncology surgeon and a caring mother.

The suggested ways to increase the feasibility for a woman to be both a successful gynecologic oncology surgeon and a caring mother are presented in **Figure 3**.

On the question: “According to you could ESGO and ENYGO help improving the gender disparity in the field of gynecologic oncology surgery?” 76.5% (62/81) of female and 55.6% (20/36) of male gave a positive answer. **Table 5** presents selected individual suggestions on options how ESGO and ENYGO could help.

**Table 5:** Selected individual suggestions on how ESGO and ENYGO could help improving the gender disparity in gynecologic oncology surgery.

•	This survey is already helpful and is a good start in promoting female to reach parity and change the perspective of the old generation.
•	Encourage and support female in their clinical and research fellowships.
•	Organize hands on courses and courses in leadership especially designed for female.
•	ESGO and ENYGO should promote female as role models at the conferences. At least 50% female at all of their committees. More female at leadership position in the both of the organizations. Also, at least 50% of conference speakers should be female.
•	Provide a better network between colleagues from different centers.
•	Successful female in this field should openly talk on conferences about managing family and work.
•	Gender disparity issues should be analyzed within ESGO and ENYGO committees. The visuality of this problem should be increased at open forums. Discussions and public awareness initiatives are needed. Promotion of activities to spread awareness of this problem.
•	Providing training scholarships and grants for female. Giving priority to applicants with children.
•	Fellowships should be well paid and should be with a shorter duration. More regional meetings should be organized. Mentor-fellow system of training in the host hospitals for better acquisition of specific surgical skills.
•	Female mentorship programs. Encouraging academic/clinical leads to facilitate and support female.
•	Providing childcare during conference, financial support for female coming to conferences with children. Providing special time and places at congress for female surgeons with children.
•	More online educational platforms.
•	Promoting a culture of understanding of mothers.
•	Promoting female surgical career and put in value the female’s work in this field.
•	Support pregnant female in being allowed to still do surgery. Setting up mentoring and fellowship programs, especially for female and encouraging hospitals to invest in the training of their female gynecologic oncology surgeons.
•	To encourage young woman to do surgery.
•	Flexible working models.
•	Day-care organized by the clinical setting.

**Figure 4:**
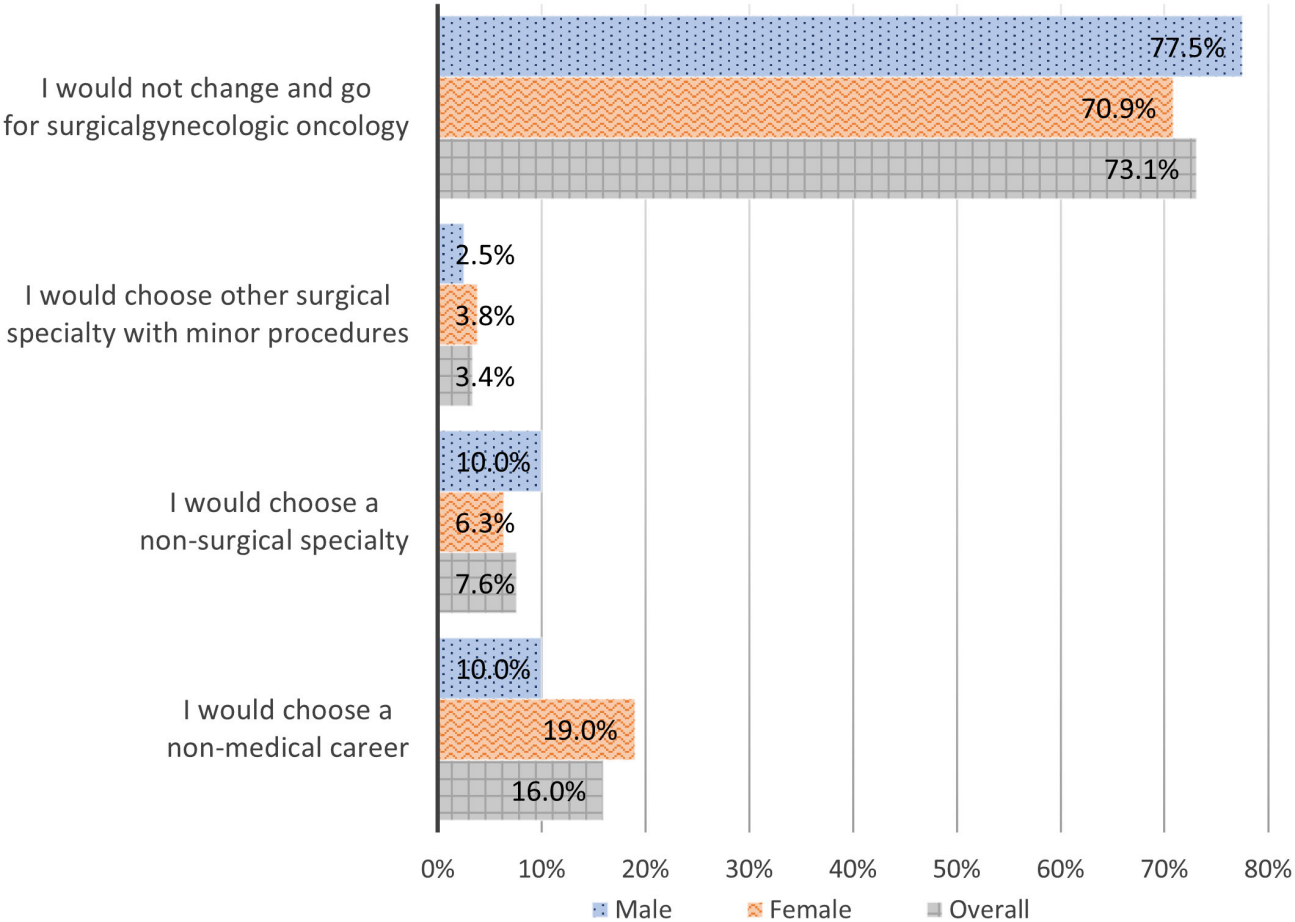
If you were now about to choose your career, what would you do?

**Figure 4** presents the answers to the question: “If you were now about to choose your career, what would you do?”. 77.5% (31/40) of male and 70.9% (56/79) of female answered that they would stay with their career choice. 10% (4/40) of male and 19% (15/79) of female stated that they would choose a non-medical career. There was not a significant association between gender and the current career choice χ^2^ (3, *N* = 119) = 2.1, *p* = .55).

## Discussion

In this study we report on gender disparities in the critical time of career development among the young generation of gynecologic oncology surgeons in Europe based on an international survey. To the best of our knowledge this is the first comprehensive evaluation of gender differences and experienced obstacles in carrier development among gynecologic oncology surgeons.

Self- reported data in our survey showed a higher exposure of male compared to female to operative procedures during their fellowships and as young specialists in gynecologic oncology surgery (**Table 2**). The predominance of male over female surgeons has been described before and is well known in different surgical disciplines, e.g. a Canadian population based retrospective study has included all surgical disciplines and reported that only 12.4% of identified surgeries were performed by females (12).

Although the majority of specialists in gynecology and obstetrics worldwide are nowadays female, the published literature reports on an underrepresentation of female in leadership positions (13, 14). The comparison of two observational studies on leadership positions in gynecology and obstetrics performed in the periods 2012/2013 and 2019/2020 in United States of America shows a slight increase in the percentage of female in leadership positions. In the period 2012/2013 20% of the chairs of departments were female; 36.1% of the vice chairs and 29.6% of the division directors (15). In the period 2019/2020 female were 29% of the chairs, 46% of the vice chairs, and 47% of the division directors (16).

Gender disparity in leadership position has been previously reported in the field of medical oncology as well. Banerjee et al. demonstrated a significant male dominance at leadership positions among respondents mainly practicing in Europe, 71% (11). Furthermore, a recent leadership study in the United States of America identified the absence of female gynecologic oncologists at cancer center director positions (17). Another study in the United States of America exposed that female constituted only a minority of all faculty in academic oncology institutions (medical oncology, radiation oncology and surgical oncology) and the low female representation was particularly pronounced at a leadership level (18).

In our study the male dominance at leadership positions was particularly displayed when focusing on leadership at younger age (under 41 years) and on younger age plus having children (**Table 3**). The adjusted odds ratio for male in a leadership position was more than 3 and notably more than 10 times higher, respectively, compared to female with these attributes in life. Parenting and domestic duties mainly carried out by female together with other factors probably contribute to hampered career advancement and gender disparity seen in leadership positions. Among high-achieving young physician-researchers it was reported that female with children spent a longer time on domestic duties and were more dedicated to childcare activities compared to male (19). Furthermore, the impact of gender and parenthood on physician’s career success was investigated by Buddeberg-Fischer B. at al. who performed a prospective study on career development among young physicians in the first seven years after graduation in Switzerland. They found out a lower career success among female physicians, especially those with children in comparison to their male colleagues. Moreover female physicians with children tended to work in smaller hospitals or private practices and aspired less often to senior hospital leading positions (20).

In our study a significantly higher proportion of female than male believed that they earn less than their gender counterparts at the same clinical position with comparable clinical and academic qualifications, with an impressive odds ratio of around 17. This estimate is leveled with data reported by Croft et al. who analyzed exact annual income sums among gynecologic oncologists in USA. They found that 75% of female gynecologic oncologists in academic settings make bellow the median salary calculated for the combined group of gynecologic oncologists of both genders (21). Further reports have robustly pointed out the gender reimbursement gap among medical oncologists in Europe and among health care providers in the USA (11, 22, 23).

With respect to PhD degrees and professorship positions, our finding in gynecologic oncology surgery are in contradiction to data reported in medical oncology by Elez et al. (24), who did report on a gender gap, while we did not reveal a gender disparity in our survey. However, our analysis showed that male published more than female. This is in line with previous gender related publication analyses in the field of gynecologic oncology (4, 25). Furthermore, our survey revealed that male with children published significantly more than female with children, which might again be related to female taking more responsibility in domestic duties and in childcare.

Planning parenting during training was of a higher importance for female than for male respondents in this survey, which is matching with published data among general gynecologists and gynecologic oncologists (26, 27). Such difference in perceived importance of family planning seems logical in light of gender specific influence on working opportunities and impact on career advancement by a decision for a family. Also, an initial career accomplishment and a subsequent child planning is feasible for male, but not for female. Fertility struggles were reported among gynecologic oncologists in United States of America with an impressive rate of 81% of females having sought infertility counseling (26). Indeed, parental leave was mainly utilized by female and covered a much longer timeframe. It was accompanied with a feeling of being underestimated by their supervisors and by the impression that parental leave has adversely affected their career, both academically and clinically due to lack of exposure to surgical procedures.

Both, male and female, recognize that there are obstacles in the career development for female in the field of gynecologic oncology surgery. The majority of male perceive the “family concerns” as the biggest obstacle for female, whereas female related their experienced carrier barriers mainly to gynecologic oncology being a closed “male club” besides the family concerns. Curiously, none of the male respondents perceived their subspecialty as a “male club”. This finding underlines the importance of more females in leadership positions in gynecologic oncology to serve as role-models, to encourage female colleagues that they can succeed, and to support them during the process of their carrier development.

Among the offered options on how to increase the feasibility for a woman to be at a same time both a successful gynecologic oncology surgeon AND a caring mother (**Figure 3**) both genders agreed that more flexible working hours and child day-care organized by the working place could be helpful in this direction.

Valuable selected individual suggestions have been received on the open question on how ESGO and ENYGO could help to overcome gender disparity in gynecologic oncology surgery (**Table 5**): children friendly conferences with organized daycare and space for mothers and children, promotion of female as role models at ESGO and ENYGO conferences, reaching parity at ESGO and ENYGO committees and among invited conference speakers, open forums with discussion on current gender issues to increase awareness and prompt support for female in their clinical and research advancement, leadership academies for female, flexible working-models and day care organized by the working place.

Regardless of the numerous exposed challenges and barriers and the significant dissatisfaction in the field of clinical gynecologic oncology surgery, the majority of female would not change and would opt again for gynecologic oncology surgery.

### Study strengths and limitations

To the best of our knowledge our study is first comprehensive evaluation of the gender-related differences among gynecologic oncology surgeons in Europe that provides an in-depth analysis of several specific problems. Also, the used qualitative methods allow a careful description of the broad spectrum of gender climate.

The biggest limitation of the study is that it is based on self-reported data. Also, there might be a selection bias with respect to those ENYGO members, who decided to fill in the survey. ENYGO is a diverse network of physicians interested in gynecologic oncology as it is the training and certification process in gynecologic oncology in various European countries. This leads to certain limitations in our study. Since this survey was sent only to ENYGO members, representing the younger generations of gynecologic oncology surgeons, our results give insights to gender issues mainly in the third and fourth decade of age only. Based on the presented data, a more focused survey on gender discrepancies is already planned to be distributed among all ESGO members, which could enable a further more profound analyses.

## Conclusion

Although female present a rising proportion in the field of gynecologic oncology surgery in Europe, male prevail over female as surgeons in operating theaters and dominate at leadership positions. Different factors related to family and childcare seem to adversely influence the clinical career advancement among female, while the effect of family planning and parenting in male seems comparably small.

It is not a lack of attractiveness or deficient wish of female to work in the field of gynecologic oncology surgery that explains the low number of females in leadership positions in this field. The majority of female would opt again for gynecologic oncology surgery. Obviously, there are obstacles in the critical time of career development that lead to a substantial attrition of female from training to leadership functions in gynecologic oncology.

ESGO and ENYGO aim to work on the implementation of measures and programs to overcome the identified obstacles, to close gender gaps, and support female to fully invest their skills, power and indispensable potential in the field of gynecological oncology surgery.

## Data availability statement

The raw data supporting the conclusions of this article will be made available by the authors, without undue reservation.

## Ethics statement

The studies involving human participants were reviewed and approved by Medical School, University in Kielce, Poland (Number: 65/2021). Written informed consent for participation was not required for this study in accordance with the national legislation and the institutional requirements.

## Author contributions

TN performed the conception and design of the study. TN, MB, NN, KZ, JK-B, and ZR designed the survey. NN organized the database. TN and NN performed the statistical analysis. TN wrote the draft of the manuscript. All authors contributed to manuscript revision, read, and approved the submitted version. NC did the senior supervision and last revision of the manuscript.

## References

[1] OECD. Health at a glance 2021: OECD indicators. Paris: OECD Publishing (2021). doi: 10.1787/ae3016b9-en

[2] Bates C Gordon L Travis E Chatterjee A Chaudron L Fivush B. Striving for gender equity in academic medicine careers: A call to action. Acad Med (2016) 91:1050–2. doi: 10.1097/ACM.0000000000001283 PMC5954825 27332868

[3] Remich R Jones R Wood CV Campbell PB McGee R. How women in biomedical PhD programs manage gender consciousness as they persist toward academic research careers. Acad Med (2016) 91:1119–27. doi: 10.1097/ACM.0000000000001253 PMC4965300 27254008

[4] Mah SJ Makkar M Huang K Anpalagan T Reade CJ Nguyen JMV. Gender imbalance in gynecologic oncology authorship and impact of COVID-19 pandemic. Int J Gynecol Cancer Off J Int Gynecol Cancer Soc (2022) 32:583–9. doi: 10.1136/ijgc-2021-003296 35304410

[5] Plank-Bazinet JL Bunker Whittington K Cassidy SKB Filart R Cornelison TL Begg L. Programmatic efforts at the national institutes of health to promote and support the careers of women in biomedical science. Acad Med J Assoc Am Med Coll (2016) 91:1057–64. doi: 10.1097/ACM.0000000000001239 PMC4957965 27191836

[6] Freund KM Raj A Kaplan SE Terrin N Breeze JL Urech TH. Inequities in academic compensation by gender: A follow-up to the national faculty survey cohort study. Acad Med J Assoc Am Med Coll (2016) 91:1068–73. doi: 10.1097/ACM.0000000000001250 PMC4965349 27276007

[7] Jagsi R Griffith KA Stewart A Sambuco D DeCastro R Ubel PA. Gender differences in the salaries of physician researchers. JAMA (2012) 307:2410–7. doi: 10.1001/jama.2012.6183 22692173

[8] Ash AS Carr PL Goldstein R Friedman RH. Compensation and advancement of women in academic medicine: is there equity? Ann Intern Med (2004) 141:205–12. doi: 10.7326/0003-4819-141-3-200408030-00009 15289217

[9] Hill EK Blake RA Emerson JB Svider P Eloy JA Raker C. Gender differences in scholarly productivity within academic gynecologic oncology departments. Obstet Gynecol (2015) 126:1279–84. doi: 10.1097/AOG.0000000000001133 PMC4868082 26551177

[10] Carr PL Gunn CM Kaplan SA Raj A Freund KM. Inadequate progress for women in academic medicine: findings from the national faculty study. J Womens Health 2002 (2015) 24:190–9. doi: 10.1089/jwh.2014.4848 PMC4363794 25658907

[11] Banerjee S Dafni U Allen T Arnold D Curigliano G Garralda E. Gender-related challenges facing oncologists: the results of the ESMO women for oncology committee survey. ESMO Open (2018) 3:e000422. doi: 10.1136/esmoopen-2018-000422 30273420 PMC6157518

[12] Wallis CJ Ravi B Coburn N Nam RK Detsky AS Satkunasivam R. Comparison of postoperative outcomes among patients treated by male and female surgeons: a population based matched cohort study. BMJ (2017) 359:j4366. doi: 10.1136/bmj.j4366 29018008 PMC6284261

[13] Heisler CA Mark K Ton J Miller P Temkin SM. Has a critical mass of women resulted in gender equity in gynecologic surgery? Am J Obstet Gynecol (2020) 223:665–73. doi: 10.1016/j.ajog.2020.06.038 32585225

[14] Wooding DJ Das P Tiwana S Siddiqi J Khosa F. Race, ethnicity, and gender in academic obstetrics and gynecology: 12-year trends. Am J Obstet Gynecol MFM (2020) 2:100178. doi: 10.1016/j.ajogmf.2020.100178 33345906

[15] Hofler L Hacker MR Dodge LE Ricciotti HA. Subspecialty and gender of obstetrics and gynecology faculty in department-based leadership roles. Obstet Gynecol (2015) 125:471–6. doi: 10.1097/AOG.0000000000000628 PMC4304882 25568998

[16] Das D Geynisman-Tan J Mueller M Kenton K. The leadership landscape: The role of gender in current leadership positions in obstetrics and gynecology departments. J Minim Invasive Gynecol (2022) 29:952–60. doi: 10.1016/j.jmig.2022.03.013 35378266

[17] Temkin SM Rubinsak L Benoit MF Chandavarkar U Hong L Berry LK. Gynecologic oncology, gender and relevant leadership in academic medicine. J Clin Oncol (2020) 38:e19056–6. doi: 10.1200/JCO.2020.38.15_suppl.e19056

[18] Chowdhary M Chowdhary A Royce TJ Patel KR Chhabra AM Jain S. Women’s representation in leadership positions in academic medical oncology, radiation oncology, and surgical oncology programs. JAMA Netw Open (2020) 3:e200708. doi: 10.1001/jamanetworkopen.2020.0708 32159809 PMC7066474

[19] Jolly S Griffith KA DeCastro R Stewart A Ubel P Jagsi R. Gender differences in time spent on parenting and domestic responsibilities by high-achieving young physician-researchers. Ann Intern Med (2014) 160:344–53. doi: 10.7326/M13-0974 PMC4131769 24737273

[20] Buddeberg-Fischer B Stamm M Buddeberg C Bauer G Häemmig O Knecht M. The impact of gender and parenthood on physicians’ careers–professional and personal situation seven years after graduation. BMC Health Serv Res (2010) 10:40. doi: 10.1186/1472-6963-10-40 20167075 PMC2851709

[21] Croft KM Rauh LA Orr JW McKinnish TR Brown J Naumann RW. Compensation differences by gender in gynecologic oncology. Gynecol Oncol (2020) 159:9. doi: 10.1016/j.ygyno.2020.06.017

[22] Desai T Ali S Fang X Thompson W Jawa P Vachharajani T. Equal work for unequal pay: the gender reimbursement gap for healthcare providers in the united states. Postgrad Med J (2016) 92:571–5. doi: 10.1136/postgradmedj-2016-134094 27528703

[23] Hughes F Bernstein PS. Sexism in obstetrics and gynecology: not just a "women’s issue". Am J Obstet Gynecol (2018) 219:364.e1–4. doi: 10.1016/j.ajog.2018.07.006 30017680

[24] Elez E Ayala F Felip E García Campelo R García Carbonero R García Donás J. Gender influence on work satisfaction and leadership for medical oncologists: a survey of the Spanish society of medical oncology (SEOM). ESMO Open (2021) 6:100048. doi: 10.1016/j.esmoop.2021.100048 33556897 PMC7872979

[25] Penn CA Ebott JA Larach DB Hesson AM Waljee JF Larach MG. The gender authorship gap in gynecologic oncology research. Gynecol Oncol Rep (2019) 29:83–4. doi: 10.1016/j.gore.2019.07.011 PMC6690427 31417953

[26] Song M Tessier K Jensen J Leonard P Geller MA Teoh D. Differences in family planning and fertility among female and Male gynecologic oncologists. Womens Health Rep New Rochelle N (2021) 2:78–84. doi: 10.1089/whr.2020.0046 PMC8080917 33937904

[27] Hill EK Stuckey A Fiascone S Raker C Clark MA Brown A. Gender and the balance of parenting and professional life among gynecology subspecialists. J Minim Invasive Gynecol (2019) 26:1088–94. doi: 10.1016/j.jmig.2018.10.020 30389582

